# Carcass characteristics and sensory analysis of Abergelle goat breed and Abergelle crossbred goat fed hay supplemented with concentrate mixture

**DOI:** 10.1093/tas/txaa149

**Published:** 2020-08-08

**Authors:** Tewodros Alemu, Alemu Dagnachew, Alemu Tsegaye

**Affiliations:** 1 Department of Animal Science, College of Agriculture, Wollo University, Dessie Campus, Dessie, Ethiopia; 2 Sekota Dry land Agriculture Research Center, Livestock Directorate Program, Sekota, Ethiopia

**Keywords:** Abergelle, Barka, carcass, cross, goat, sensory

## Abstract

The experiment was conducted using 36 intact yearling males of Abergelle breed and Abergelle cross bred goats (50%) with initial live weight of 18.92 ± 0.72 kg (mean ± SE). The objective of the experiment was to evaluate the effect of concentrate supplementation on carcass parameters and meat sensory quality of genotypes. Goat genotypes were blocked based on initial body weight and were randomly assigned to dietary treatments. The experimental design was 2 by 3 factorial in randomized complete block design. The treatments included local grass hay as basal diet and supplementation with concentrate (184, 368, and 552 g/d on DM basis). Effects of genotype and diet were significant on the main carcass parameters (*P* < 0.05) but genotype did not show effect on edible offal components (*P* > 0.05). Diet had a significance effect on meat flavor (*P* < 0.05) but not on tenderness, juiciness, and soup flavor (*P* > 0.05). Genotype had no effect (*P* > 0.05) on all sensory attributes. Goats feeding on higher level of concentrate had heavier total edible offal components (*P* < 0.05) than feeding on lower level of concentrates but not difference between genotypes (*P* > 0.05). The cross breed goats feeding on higher level of concentrate showed higher percentage of nonedible offal (*P* < 0.01) particularly gut content, foreleg, and hind leg than pure breed and lower level of concentrate. The digestibility and chemical composition of meat of the genotypes were not addressed in the experiment and hence need to be studied further.

## INTRODUCTION

Sekota district is known by its goat production potential and the inhabitants of the area are very familiar with the consumption of goat meat compared with other ruminants. As the result of this, the regional government has categorized the area to specialize on goat production (BoA, 2003). To enhance the productivity of Abergelle goat breed, Barka goat breed, which are known in their fast growth and high milk yield, have been introduced in low land areas of Sekota district. Belay and Bewketu (2010) noted similar growth performance between Barka*Abergelle cross (50%) and pure Abergelle goat breed under on-farm conditions feeding on a natural pasture. In most cases under on-farm conditions, the energy and protein availability in the natural pasture may be not enough to meet the requirements of goats to reach high levels of growth. To reach high levels of growth, goats are usually supplemented with concentrate feeds when an improvement in growth rate is desired. However, in the similar way, Amare et al. (2018) also reported under on station the pure Abergelle and Abergelle*Barka cross bred goats had a similar biological performance when supplemented with 184, 368, and 552 g/d of commercial concentrates.

However, the two genotypes have not been characterized in terms of carcass quality and have not been compared under different feeding regimes. In addition, research was not undertaken on the effect of concentrate supplementation on the carcass characteristics and sensory analysis of meat for both genotypes, so far although sensory diversity is an important factor in consumer attitudes towards meats (Sanudo et al. 2007). Studies indicated that changes in feeding regime could modify the intrinsic characteristics of goat meat (Casey and Webb, 2010) and similarly different feeding regimes modify the taste and flavor of meat (Madruga and Bressan, 2011). On the other hand, farmers and extension workers of the study area perceive that Barka*Abergelle cross goats have lower sensory attribute (specifically in flavor and taste) and have low meat quality than pure Abergelle goats (ZAD, 2011).

Therefore, this study was proposed to determine the perception of farmers and extension workers on the sensory attributes and to evaluate the major meat carcass characteristics under different feeding regimes of the goat genotypes. The study was aimed to determine the carcass characteristics and sensory attributes, and to evaluate the effect of different levels of concentrate mixture supplementation on carcass characteristics and sensory attribute of meat of pure Abergelle breed and Abergelle*Barka cross bred goats consuming local grass hay as basal diet and supplemented with different levels of concentrate mixture.

## MATERIALS AND METHODS

The study was conducted in Sekota Dryland Agricultural Research Center, Sekota (Ethiopia). All experimental methods, particularly the treatment of animals during the trial and at slaughter, were in accordance with the guidelines of the EU Directive 2010/63/EU for animal experiments.

### Experimental Animals and Their Management

A total of 36 intact yearlings experimental goats, 18 pure Abergelle breed goats, and 18 Abergelle*Barka cross breed goats (50% of each genotype) were purchased from the market, with age based on dentition and the information obtained from the owners (Amare et al. 2018). During the quarantine period, animals were deworming with a broad spectrum antihelemantic (Albendazole), sprayed with accaricide (diazzinole), and vaccinated against anthrax and pasteurelosis.

### Feeds and Feeding Management

Local hay was purchased from the farmers and manually chopped to a size of 3–4 cm to minimize selective feeding. The local grass hay was composed of *Cynodon dactylon* (Locally called *Serdo*), *Hyperrhenia rufa* (Locally called *yebetkidan sar*), and *Guizotia abyssinica* (Locally called *Senbelet*) grass species (Amare et al., 2018). The concentrate feed was formulated by Akaki animal feed production private limited company which is located in Addis Ababa (Ethiopia). Local grass hay was fed at a rate of 20% refusal of the previous day offer to ensure ad libitum feeding (Amare et al., 2018). The supplementary concentrate feeds were 184, 368, and 552 g/d on dry matter basis (Amare et al., 2018). All goats had free access to water and common salt (NaCl). The feeding trial lasted 105 d including the 15-d adaptation period. The chemical compositions of the local grass hay and concentrate mixtures are indicated in Table 1.

**Table 1.** Chemical compositions of local grass hay and concentrate mixtures (g/kg)

Type of feed	DM	OM	CP	NDF	ADF	ASH
Local grass hay	900	800	79	620	420	100
Concentrate mix	920	830	159	236	170	90

DM = dry matter; OM = organic matter; CP = crude protein; NDF = neutral detergent fiber; ADF = acid detergent fiber.

### Experimental Design and Treatments

The experimental treatment arrangement was a 2*3 factorial in a randomized complete block design (RCBD) with six replications (Amare et al., 2018). The first factor had two levels which were Abergelle pure breed and its crossbreed (50% Abergelle and 50% Barka). The second factor had three levels which were the supplementation levels (184, 368, and 552 g/d) of concentrate (Amare et al., 2018).

### Carcass Analysis

For the carcass analysis, the experimental goats were fasted overnight, weighed, and slaughtered. The goats were killed by severing the jugular vein and the carotid artery. The blood was drained into bucket and weighted. The gastro-intestinal tract with the exception of the esophagus was removed with its contents and weighed. The gastro-intestinal organs were reweighed after emptying its contents. Fat in gastro-intestinal tract and kidney was individually weighed. After evisceration, the hot carcass was weighed and cut perpendicular to the back bone between the 12th and 13th ribs to measure the cross-sectional area of the rib eye muscle area (Purchas 1978). The rib eye area was traced first on transparency paper and measured using mechanical polar plani-meter. The empty body weight was calculated as gut content deducted from slaughter weight. Dressing percentage was calculated as proportion of hot carcass weight, as empty body weight and slaughter body weight bases.

Generally for the carcass characteristics, the parameters measured were slaughter weight, hot carcass weight, empty body weight, dressing percentage as slaughter body weight basis (SW), dressing percentage as empty body weight basis (EBW), thin cuts, fore shank, neck and shoulder, ribs, hind shank, loin, and rib eye muscle area. For the weight of edible offal component (EOC) measured were head with tongue, heart, liver, kidney, empty stomach, tail, abdominal fat, kidney fat, testes, small intestine, large intestine, blood, total edible offal content, edible offal percentage, and total usable product percentage. Similarly, the components measured for the nonedible offal were skin, fore leg, hind leg, lung and trachea, spleen, penis, gall bladder, gut content, total nonedible offal component (TNEOC), and percentage of nonedible offal.

### Sensory Analysis

For the sensory analysis, meat samples were taken from loin, ribs, and legs from each experimental goat for comparison. A soup was also prepared from meat cuts of loin, ribs, and legs. The samples were then presented randomly between panelists and sessions. Nine panelists who were very familiar with goat meat performed their evaluations in individual booths in preadjusted room prepared for this purpose.

The fresh meat portions were cut into pieces of small cubes of equal size approximately 2.5 cm^3^ before cooking. The firing was done in a convection oven until reaching 80 °C at the heart of the product for approximately 5 min and offered to the panel of evaluators. During a preliminary phase, discussions were held on the sensory expressions to avoid doubt about the meaning of attributes. A profile protocol was developed relating to meat tenderness (extremely tender, very tender, moderately tender, tough, very tough); meat juiciness (extremely juicy, very juicy, moderately juicy, slightly juicy, very tough); meat flavor (extremely intense, very intense, moderately intense, slightly intense and bland); and flavor of meat soup (extremely intense, very intense, moderately intense, slightly intense and bland) to be was used for the evaluation.

The tenderness, juiciness, and flavor of meat were evaluated for the different portions of the meat (loin, ribs, and legs) of each sample separately and the average judges or scores of the assessors were taken. But, the soup flavor was evaluated by cooking the different portions of meats together. Every panelist assessed the meat samples prepared from the 6 treatments which had 6 replications for the 4 different attributes (meat tenderness, meat juiciness, meat flavor, and soup flavor). The panelists were instructed to rinse their mouth by eating a piece of bread and to drink a sip of distilled water at the beginning of the sensory evaluation and between sample trials to make the palate conditions similar for each sample.

### Statistical Analysis

Data on the carcass parameters of slaughter weight, hot carcass weight, empty body weight, dressing percentage (as slaughter body weight basis), dressing percentage (as empty body weight basis), thin cuts, fore shank, neck and shoulder, ribs, hind shank, loin, and rib eye muscle area were subjected to analysis of variance and analyzed by using the general linear model procedure of SAS (2002). Similarly, the data collected on the edible and nonedible offal: head with tongue, heart, liver, kidney, empty stomach, tail, abdominal fat, kidney fat, testes, small intestine, large intestine, blood, total edible offal content, edible offal percentage, percentage of total usable product, skin, fore leg, hind leg, lung and trachea, spleen, penis, gall bladder, gut content, total nonedible offal content, and percentage of non-edible offal were analyzed by using the general linear model procedure of SAS. Mean separation was done by Duncan’s multiple range Test (Duncan, 1955) and significance thresholds of 0.05, 0.01, and 0.001 were used. Since the interaction effect of the treatments was never significant in all traits, it was not tabulated in the results.

Chi-square test was applied to evaluate if genotype of goats and feeding levels affected the frequency distribution of consumer preferences for the different goat meat types on acceptability information obtained from each consumer. The test was made for replies of assessors on the tenderness, juiciness, and flavor of meat. Similarly, test was made for the replies of assessors on flavor of the soup.

## RESULTS

### Carcass Component

Dressing percentage as slaughter weight and dressing percentage as empty body weight for the cross bred goat and thin cuts for the pure breed goat showed significant difference among the diets (*P* < 0.05). However, the rest carcass parameters did not show significant difference among the diets (*P* > 0.05).

On the other hand, genotype showed significant difference on slaughter weight (*P* < 0.05), dressing percentage as slaughter weight (P < 0.05), hot carcass weight (*P* < 0.001), and empty body weight (P < 0.001). But, the other traits of carcass characteristics did not show significant difference between the genotypes (*P* > 0.05). Carcass characteristics of Abergelle and Abergelle*Barka cross bred goats is shown in Table 2.

**Table 2.** Carcass characteristics of Abergelle and Abergelle*Barka cross bred (50%) goats

Variables	Genotype	Supplementation level			SE	*P*	
		184 g/d	368 g/d	552 g/d		Diet	Genotype
Slaughter weight, kg	Abergelle	20.1	24.8	22.6	3.52	NS	*
	Cross	22.6	29.4	27.4		NS	
Hot carcass weight, kg	Abergelle	8.4	10.9	9.8	3.52	NS	***
	Cross	9.1	12.6	10.8		NS	
Empty body weight, kg	Abergelle	16.6	21.1	19.2	2.50	NS	***
	Cross	18.1	24.6	22.98		NS	
Dressing %, SW	Abergelle	41.3	43.9	43.5	3.53	NS	*
	Cross	40.4^b^	42.8^a^	39.6^b^		*	
Dressing %, EBW	Abergelle	50.3	51.7	51.3	3.52	NS	NS
	Cross	50.6^a^	51.1^a^	47.3^b^		*	
Thin cuts, kg	Abergelle	0.4^b^	0.7^a^	0.6^ab^	3.52	*	NS
	Cross	0.47	0.63	0.59		NS	
Fore shank, neck, and shoulder, kg	Abergelle	2.6	3.66	3.53	2.49	NS	NS
	Cross	3.2	4.27	3.87		NS	
Ribs, kg	Abergelle	2.03	2.53	2.43	3.52	NS	NS
	Cross	2.23	3.17	2.6		NS	
Hind shank, kg	Abergelle	2.5	3.23	3.06	2.50	NS	NS
	Cross	2.87	3.67	3.4		NS	
Loin, kg	Abergelle	0.46	0.67	0.61	3.52	NS	NS
	Crosss	0.53	0.75	0.64		NS	
Rib eye muscle area, cm^2^	Abergelle	7.56	8.5	8.45	3.52	NS	NS
	Cross	7.48	8.89	8.38		NS	

Means in the same row with different superscripts differ significantly.

*P < 0.05; **P < 0.01; ***P < 0.001; NS = not significant.

### Offal Component

In Ethiopia, offal is categorized into edible and nonedible components based on the culture of the people in different parts of the country. This classification was based on the study area’s meat consumption cultural practice.

#### Edible offal component.

Testis and total edible offal components in the pure breed goat and liver in cross bred goat were significantly different (*P* < 0.05) among diets. However, most edible offal components were not affected by diet (*P* > 0.05).

On the other hand, all edible offal components were not affected by genotype (*P* > 0.05). The edible offal of Abergelle goats breed and their cross bred goats is reported in Table 3.

**Table 3.** Edible offal of Abergelle goats breed and their cross bred (50%) goats

Variables	Genotype	Level of supplementation			SE	*P*	
		184 g/d	368 g/d	552 g/d		Diet	Genotype
Head with tongue, g	Abergelle	1280	1430	1300	30.5	NS	NS
	Cross	1460	1750	1570		NS	
Heart, g	Abergelle	100	120	130	30.5	NS	NS
	Cross	120	150	170		NS	
Liver, g	Abergelle	280	370	380	30.5	NS	NS
	Cross	280^b^	400^a^	440^a^		*	
Kidney, g	Abergelle	57	70	72	30.5	NS	NS
	Cross	73	82	79		NS	
Empty stomach, g	Abergelle	687	707	705	30.5	NS	NS
	Cross	710	910	900		NS	
Tail, g	Abergelle	59	74	73	30.5	NS	NS
	Cross	80	77	78		NS	
Abdominal fat, g	Abergelle	180	650	430	21.0	NS	NS
	Cross	210	390	540		NS	
Kidney fat, g	Abergelle	23	76	22	30.5	NS	NS
	Cross	55	41	94		NS	
Testes, g	Abergelle	90^b^	180^a^	150^a^	30.5	*	NS
	Cross	140	180	190		NS	
Small intestine, g	Abergelle	639	653	654	30.5	NS	NS
	Cross	580	770	920		NS	
Large intestine, g	Abergelle	90	80	100	30.5	NS	NS
	Cross	110	99	115		NS	
Blood, g	Abergelle	780	980	1060	30.5	NS	NS
	Cross	970	1120	1150		NS	
Total EOC, kg	Abergelle	4.26^b^	5.65^a^	5.15^ab^	30.5	*	NS
	Cross	5.12	6.14	6.28		NS	
Edible offal, %	Abergelle	21.23	22.86	22.84	30.5	NS	NS
	Cross	21.55	20.73	21.49		NS	
Total usable product, %	Abergelle	62.53	66.72	66.36	30.5	NS	NS
	Cross	63.05	63.77	62.13		NS	

Means in the same row with different superscripts differ significantly.

*P < 0.05; **P < 0.01; ***P < 0.001. NS = Not significant.

#### Nonedible offal component.

Nonedible offal percentage in the pure and cross breed goats and spleen only in the pure breed goat was significantly different among the diets (*P* < 0.05). But, the other nonedible offal components were not significantly affected (*P* > 0.05) by diet.

The genotypes showed significantly different in gut content (*P* < 0.01), foreleg (*P* < 0.01), and hind leg (*P* < 0.05). But, most of the other nonedible offal components were not significantly affected (*P* > 0.05) by genotype. The nonedible offal component of Abergelle and the cross (50%) goats is indicated in Table 4.

**Table 4.** Nonedible offal component of Abergelle and the cross (50%) goats

Variables	Genotype	Level of supplementation			SE	*P*	
		184 g/d	368 g/d	552 g/d		Diet	Genotype
Skin, g	Abergelle	1310	1700	1570	151	NS	NS
	Cross	1470	1710	1850		NS	
Fore leg, g	Abergelle	800	310	290	151	NS	**
	Cross	320	420	390		NS	
Hind leg, g	Abergelle	240	250	260	151	NS	*
	Cross	290	350	310		NS	
Lung and trachea, g	Abergelle	280	250	230	151	NS	NS
	Cross	260	360	280		NS	
Spleen, g	Abergelle	30	40	50	151	*	NS
	Cross	40	70	60		NS	
Penis, g	Abergelle	60	40	70	151	NS	NS
	Cross	70	80	80		NS	
Gall bladder, g	Abergelle	10	10	10	107	NS	NS
	Cross	20	10	20		NS	
Gut content, g	Abergelle	3570	3730	3430	151	NS	**
	Cross	4530	4820	4430		NS	
TNEOC, kg	Abergelle	5.91	6.37	6.15	151	NS	NS
	Cross	6.99	7.83	7.43		NS	
Nonedible offal, %	Abergelle	29.49^a^	25.7^b^	27.12^a^	151	*	NS
	Cross	32.49^a^	26.97^b^	28.66^b^		*	

Means in the same row with different superscripts differ significantly.

*P < 0.05; **P < 0.01; ***P < 0.001; NS = Not significant.

### Sensory Evaluation

The chi-square test showed that there was a significant difference (*P* < 0.05) among the feed levels in meat flavor of the goats. However, significant differences were not observed in the other sensory parameters (*P* > 0.05) among the feed levels and in all sensory parameters between the genotypes. Sensory evaluation of meat from pure Abergelle and Abergelle*Barka (50%) cross goats is indicated in Table 5.

**Table 5.** Sensory evaluation of meat and meat soup from Abergelle and Abergelle*Barka (50%) goats

Variables	Score	Feeding level			Genotype	
		184 g/d	368 g/d	552 g/d	Abergelle	Cross
Tenderness of meat, %	Extremely tender	11.1	11.1	25.9	16.1	18.5
	Very tender	25.9	25.9	37	29.6	32.1
	Moderately tender	40.7	37	33.3	38.3	30.9
	Tough	18.5	25.9	3.7	16.1	16.1
	Very tough	3.7	0	0	1.23	2.5
*P* = 0.29		NS			NS	
Juiciness of meat, %	Extremely juicy	7.4	7.4	7.4	7.4	4.9
	Very juicy	7.4	7.4	22.2	12.4	22.2
	Moderately juicy	37.1	25.9	25.9	29.6	30.9
	Slightly juicy	44.4	48.2	29.6	40.7	32.1
	Very tough	3.7	11.1	14.8	9.9	9.9
*P* = 0.53		NS			NS	
Flavor of meat, %	Extremely intense	11.1	11.1	18.5	13.6	14.8
	Very intense	25.9	29.6	48.1	34.6	35.8
	Moderately intense	25.9	48.2	29.6	34.7	33.3
	Slightly intense	29.6	11.1	3.7	14.8	12.4
	Bland	7.4	0	0	2.5	3.7
*P* = 0.044		S			NS	
Flavor of soup, %	Extremely intense	11.1	0	33.3	14.8	14.8
	Very intense	44.4	44.4	33.3	40.7	33.3
	Moderately intense	33.3	33.3	22.2	29.6	29.6
	Slightly intense	11.1	22.2	11.1	14.8	22.2
	Bland	0	0	0	0	0
*P* = 0.62		NS			NS	

Means in the same row with different superscripts differ significantly.

*P < 0.05; **P < 0.01; ***P < 0.001; NS = Not significant; S = Significant.

## DISCUSSION

### Carcass Component

The cross bred genotype showed significantly higher than pure breed in slaughter weight, hot carcass weight, and empty body weight. But, the dressing percentage as slaughter weight was significantly higher for pure breed than the cross bred goat. On the other hand, dressing percentage as empty body weight of the pure breed was similar with the crossbred. The supplementation level resulted in up and down in the carcass traits. This could be due to differences between goats in the different groups in noncarcass components. The higher dressing percentage as slaughter weight for pure breed but similar as empty body weight indicates that the cross bred goats may have high content of gut fill. The research result of the two genotypes is comparable with findings of Sidama goat with dressing percentage of 47.7–55.5% (Megersa et al., 2012). However, the genotypes seem to be less efficient in carcass production compared with Somali goat breed with dressing percentage of 56.99–57.7% (Betsha and Solomon 2008).

### Offal Component

According to Adisu et al. (2001), any goat breed evaluation studies for meat production should emphasize the need to pay attention to the total yield of usable products, rather than only the carcass weight and dressing percentage, particularly in cultures where edible offal components are traditionally consumed.

#### Edible offal component.

The testis and total edible offal components of the pure breed goat feeding on the higher level of concentrate had heavier in weight than those feeding on lower level of concentrate. Similarly, the liver of cross bred goats feeding on the higher level of concentrate had heavier in weight than those feeding on lower level of concentrate. The increase in weight with high concentrate ratio might be related to the storage of reserve carbohydrates such as glycogen when animals are fed with energy dense diets (Lawrence and Fowler, 1998). The result is in contrast to the report of Saikia et al. (1996) who indicated that different feed supplements had no effect on edible offal due to the growth phase of the goats.

#### Nonedible offal component.

Generally most nonedible offal components are said to be early maturing and lack of significant effect of diet for most may be expected. However, in the present study, diet had an effect on nonedible offal percentage in both pure and cross breed goats.

The cross breed goats showed higher gut content than the pure breed goat. The average gut fill ranged from 15.1 to 20% of live weight in different dietary groups that correspond to the findings of Betsha (2005) who found gut fill to be 14.95 to 23.58% of live weight. The cross breed goats also showed higher foreleg and hind leg. These might be due to the framework of the cross bred goat, i.e., the appendixes and the belly of the cross are longer and wider than the pure breed, respectively. The result is in contrast to the report of Saikia et al. (1996) who indicated that feed supplement had no effect on nonedible offal due to the growth phase of the goats.

### Sensory Evaluation

Sensory diversity is an important factor in consumer attitudes towards meats (Sanudo et al. 2007). Analysis of variance in the sensory parameters of meat revealed a significant difference among the feed levels in meat flavor of the goats. Feeding higher level of concentrate (368 and 552 g/d) resulted in better flavor of meat, but genotype did not show significant effect on the flavor of meats. The result indicates that the level of supplementation significantly affects the flavor of meats although development of flavor of meat is a very complex system (Piasentier et al. 2009). However, this result is in contrast with the farmer belief that meat from Abergelle goat is superior eating qualities than cross bred goat.

On the other hand tenderness, juiciness and soup flavor of the meats did not show significant differences among feeding levels and between the genotypes. The result is similar with the comment made by Moloney et al. (2001) that feeding pattern and ration composition generally have little impact on the tenderness of meat in cattle but in contrast to the report of Maltin (2003) who indicated breed and nutrition may play a part in determining the tenderness of meat.

## CONCLUSION

The effects of genotype and diet were significant on some of the main carcass parameters, but genotype did not show significant effect on the edible offal components. Diet had a significance effect on meat flavor but not on tenderness, juiciness, and soup flavor. Genotype had no effect on all sensory attributes. Goats feeding on the higher level of concentrate had heavier total edible offal components than feeding on lower level of concentrates but not difference between the genotypes. The cross breed goats feeding on higher level of concentrate showed higher percentage of nonedible offal, particularly gut content, foreleg, and hind leg than the pure breed and lower level of concentrate. In the sensory analysis, diet has shown a significant effect on meat flavor. The digestibility and chemical composition of meat of the goats were not addressed in the experiment and hence need to be studied further.
